# The effects of maribavir on the autophosphorylation of ganciclovir resistant mutants of the cytomegalovirus UL97 protein

**DOI:** 10.1186/2042-4280-1-4

**Published:** 2010-12-07

**Authors:** Claire D Shannon-Lowe, Vincent C Emery

**Affiliations:** 1 Department of Infection, Centre for Virology, UCL (Royal Free Campus Campus), Rowland Hill Street, Hampstead, London NW3 2QG, UK

## Abstract

**Background:**

The UL97 protein kinase of human cytomegalovirus phosphorylates the antiviral drug ganciclovir and is the target of maribavir action. A detailed enzyme kinetic analysis of maribavir on the various enzymatic functions of wild type and ganciclovir resistant forms of UL97 is required.

**Methods:**

Wild type and site directed mutant forms of the human cytomegalovirus UL97 gene product were expressed using recombinant baculoviruses and the purified products used to assess the effects of maribavir on the ganciclovir (GCV) kinase and protein kinase (PK) activities.

**Results:**

Maribavir was a potent inhibitor of the autophosporylation of the wild type and all the major GCV resistant UL97 mutants analysed (M460I, H520Q, A594V and L595F) with a mean IC_50 _of 35 nM. The M460I mutation resulted in hypersensitivity to maribavir with an IC_50 _of 4.8 nM. A maribavir resistant mutant of UL97 (L397R) was functionally compromised as both a GCV kinase and a protein kinase (~ 10% of wild type levels). Enzyme kinetic experiments demonstrated that maribavir was a competitive inhibitor of ATP with a Ki of 10 nM.

**Discussion:**

Maribavir is a potent competitive inhibitor of the UL97 protein kinase function and shows increased activity against the M460I GCV-resistant mutant which may impact on the management of GCV drug resistance in patients.

## Background

The UL97 gene of human cytomegalovirus encodes a 690 amino acid nuclear serine/threonine protein kinase that is present in the virion and is critical for efficient viral replication [1-8]. The precise function of UL97 in the replication cycle has not been fully elucidated but UL97 has been implicated in replication, viral DNA packaging, nuclear egress, virion morphogenesis and cell cycle manipulation [9-15]. The full range of targets for UL97 kinase action are also undefined although the viral UL44 protein is one viral substrate [16] and pUL97 complexes with the tegument phosphoprotein pp65 (ppUL83)[17]. UL97 also phosphorylates the antiviral nucleosides ganciclovir (GCV) and acyclovir [18-21], the former being the major drug currently used to control HCMV replication in vivo [22,23]. In the era preceding the deployment of highly active antiretroviral therapy for HIV infection, long term GCV therapy for cytomegalovirus retinitis led to the development of GCV resistance in up to 40% of patients [24] although the frequency appears to be lower in transplant recipients receiving GCV prophylaxis [24-27]. A number of studies have investigated the molecular basis for resistance of HCMV to GCV and show that a small number of mutations in UL97 (at amino acids M460, H520, A594 and L595) account for the majority (~85%) of clinically significant drug resistance [28-31]. Subsequent to the development of UL97 mutations, resistance at the DNA polymerase locus can also occur [32-34]. In vivo dynamic modelling has shown that these mutant viruses are less fit than their wild type counterparts and, in the absence of selective drug pressure, wild type virus is between 5-10% more fit than the GCV resistant mutants [35].

The benzimidazole class of compounds are novel inhibitors of HCMV replication. The progenitor, BDCRB (2-bromo-5,6-dichloro-1-(β-D-ribofuranosyl benzimidazole) inhibits the packaging of HCMV genomes via the UL89 and the UL56 terminase proteins [36] whereas the related compound maribavir (5,6-dichloro-2-(isopropylamino)-1-beta-L-ribofuranosyl-1H-benzimidazole; also known as 1263W94 and benzamidavir) targets the protein kinase activity of UL97 (37). Maribavir has potent antiviral activity against HCMV [37] and EBV [38], with an IC_50 _approximately 4-to 10-fold lower than that observed for GCV against HCMV replication in vitro and is active against HCMV strains resistant to ganciclovir, foscarnet and cidofovir [39]. Cell culture of HCMV in the presence of maribavir routinely leads to drug resistance involving a single amino acid change in UL97: L397R [37]. However, recently, using a clinical isolate with a mutation in the exonuclease domain of the viral DNA polymerase additional UL97 mutations at amino acids 411 (H to L/Y/N), 409 (T to M) and 353 (V to A) have also been described [40,41]. These mutations cluster more closely to the proposed ATP binding domain of UL97. Maribavir has undergone phase I and II clinical trials and phase III prophylaxis trials in solid organ and stem cell transplant recipients [42-44]. Mutations which contribute to drug resistance against maribavir have also been identified in UL27 although the precise function of this protein in the viral life cycle remains to be fully defined [45].

At present, a detailed enzyme kinetic analysis of drug resistant UL97 mutants has not been undertaken. In the present study, we have used recombinant baculovirus systems to produce UL97 protein of high purity to perform a detailed biochemical analysis of the kinetics of autophosphorylation of a series of the clinically relevant UL97 GCV resistant mutants and a selection mutations in conserved protein kinase domains in UL97 to determine the effects of maribavir on the autophosphorylation capacity of these mutants. The data provide insight into the mechanism of action of maribavir and highlight the impact that certain GCV resistant mutations have on the inhibitory profile of maribavir.

## Methods

### Cells and viruses

Spodoptera frugiperda 21 (Sf21) cells (Invitrogen) were maintained in TC100 medium (Life Technologies, Paisley Scotland) supplemented with 10% fetal bovine serum, 50 IU of penicillin per ml, and 50 μg of streptomycin per ml. Wild-type linearized Autographa californica multiple nuclear polyhedrosis virus (AcMNPV; Invitrogen, Scotland) was used to construct the recombinant baculoviruses. The Towne strain of cytomegalovirus was propagated in primary human embryonic lung fibroblasts maintained in DMEM medium (Life Technologies, Scotland) supplemented with 10% fetal calf serum, 2 mM L-glutamine, 100 IU of penicillin per ml, and 100 μg of streptomycin per ml in 5% CO2.

### Cloning of the HCMV UL97 ORF

Human embryonic lung fibroblasts were infected with the Towne strain of HCMV at a multiplicity of infection (m.o.i.) of 5 and 72 h later DNA extracted using the Wizard genomic purification kit (Promega, Southampton UK). The extracted DNA (1 ug) was used as the template for a PCR reaction to generate the full length UL97. The primers (5'-GGG GTA CCC ATG TCC TCC GCA CTT CGG TCT CGG and 5'-CCC AAG CTT TTA CTC GGG GAA CAG TTG GCG GCA) were designed to generate an amplicon with a 5' Kpn I and a 3' Hind III restriction site. PCR conditions consisted of 95°C for 5 min followed by cycling at 60°C for 3 min, 72°C for 10 minutes and 95°C for 3 minutes using the Bioline (UK) high fidelity Taq polymerase mixture (Bio-X-Act) according to the manufacturer's instructions. The 2160 bp DNA amplicon was purified using the Wizard™PCR prep DNA purification system (Promega, UK) and ligated into the pGEM-T Easy vectors (Promega, UK) for subsequent sequencing of the entire UL97 ORF. The pGEM-UL97 construct was restricted with Kpn I and Hind III, the UL97 fragment purified and ligated into the pMelBac C or pBluBacHis C transfer vectors (Invitrogen, Scotland), which had been similarly restricted enabling directional cloning of the UL97 in the correct open reading frame. The pMelBac transfer vector allowed the incorporation of a N-terminal honey bee melittin secretion signal onto the UL97 protein while the pBluBacHis incorporated an N-terminal hexa-histidine tag.

### Site directed mutagenesis of the UL97 ORF

Point mutations were introduced into the pMelUL97 transfer vectors using the GeneEditor™in vitro Site Directed Mutagenesis system (Promega, Southampton, UK). Briefly, 100 ng of denatured template DNA was added to an annealing solution of 0.25 pmol of the appropriate phosphorylated selection oligonucleotide (top strand), 1.25 pmol of phosphorylated mutagenic oligonucleotide and 10 × TM buffer (100 mM Tris, 100 mM MgCl_2_). Annealing was performed for 15 min at 37°C and mutant strand synthesis and ligation performed at 37°C for 90 min following addition of 10 × synthesis buffer (100 mM Tris-HCl, pH7.5, 5 mM dNTP, 10 mM ATP, 20 mM DTT), T4 DNA polymerase, and T4 DNA ligase according to manufacturers instructions. Following the mutagenesis reaction, 1.5 μl of each reaction was transformed into BMH 71-18 *mutS E.coli *, the plasmid harvested and transformed into JM109 *E.coli *in an appropriate antibiotic selection mix to ensure good segregation of the mutant and wild type plasmids. The mutant UL97 ORFs were DNA sequenced to confirm the specific introduction of the mutation and the absence of other mutations and recombinant baculoviruses generated as described below. The primers used to introduce the desired mutations were as follows: K355Q (5'-CGC GTG GTC GAG GTG GCG CG); L397R (5'-CGC GGT CTG CGC ACG GCC AC); D456A (5'-TGC CAC TTT GCC ATT ACA CCC); M460I (5'-C ATT ACA CCC ATT AAC GTG C); N461G (5'-ACA CCC ATG AAA GTG CTC ATC); H520Q (5'-GAA TGT TAC CAG CCT GCT TTC C); A594T (5'-C TGC CGC ACG TTG GAG AAC GG); L595F (5'-C TGC CGC GCG TTT GAG AAC GG); H662L(5'-ACC ATG CTG CTC GAA TAC GTC) and V665I (5'-CAC GAA TAC ATC AGA AAG AAC G).

### Production of recombinant baculoviruses expressing wild type and mutant forms of UL97

Recombinant baculoviruses were constructed by co-transfection of the transfer vectors with linear, wild type AcMNPV DNA into Sf21 cells using InsectinPlus™liposomes (Invitrogen, Scotland). Recombinant baculoviruses BVBMUL97 or BVBBHUL97 were then harvested 72 hours post transfection and expanded by two rounds of plaque purification to high titre stocks. Insertion of the UL97 and absence of the wild type AcMNPV were confirmed by PCR using primers either side of the cloning sites. Expression of the wild type or mutant UL97 proteins was assessed using SDS-PAGE and Western blotting following infection of Sf21 insect cells at an m.o.i. of 10. After 72 h, cells were lysed and proteins were separated by SDS-PAGE and electrophoretically transferred onto a polyvinylidene difluoride (PVDF; Bio-Rad) membrane by using a semi-dry blotter (Pharmacia Biotech, Buckinghamshire, UK). The membrane was incubated for 1 h at room temperature in blocking buffer (3% bovine serum albumin (Sigma Chemicals, Dorset, UK) in TBS [10 mM Tris-HCl, pH 7.5, 150 mM NaCl]), then washed with 0.05% Tween 20-0.1% Triton X-100 in TBS. The membrane was incubated with a 1/500 dilution of mouse UL97-specific polyclonal antisera (a gift from Dr D Michel, Universitatsklinikum Ulm, Abteilung Virologie, Ulm, Germany) in blocking buffer for 1 h at room temperature then incubated with 1:6,000 dilution of alkaline phosphatase-conjugated goat anti-mouse IgG (Bio-Rad, Hemel Hempstead, UK) for 1 h at room temperature. The membrane was further washed, and immunoreactive bands were visualized by incubation of the membrane in a staining solution consisting of one tablet of 5-bromo-4-chloro-3-indolylphosphate-nitroblue tetrazolium chloride (Sigma Chemicals, Dorset, UK) dissolved in 10 ml of distilled H_2_O.

### Purification of the wild type and mutant UL97 proteins

Sf21 insect cells were infected with wild type or mutant UL97 recombinant baculoviruses at an m.o.i. of 10, incubated at 28°C and harvested 72 hours post infection. Cells were sonicated three times in ice cold sonication buffer (50 mM Tris-HCl, pH 7.6, 100 mM NaCl, 0.1% Nonidet P-40, 10% glycerol, 10 μg/ml Aprotinin and Leupeptin and 1 mM Pefabloc), for 10 seconds at 5 microns. Disrupted cells were centrifuged for 10 minutes at 4°C at 13000 × g. Ammonium sulphate was added to the sonicated crude lysate to give a% saturation of 20%-40%, at which the maximum concentration of UL97 was precipitated. The protein precipitate was centrifuged at 3000 × g for 40 minutes at 4°C, resuspended in 20 mM Tris-HCl, pH 8.5, 100 mM NaCl, 0.5% Sarkosyl and dialysed overnight to give a total of 1/10 000 dilution of the ammonium sulphate. The insoluble proteins were removed by centrifugation and the UL97 dialysed into 20 mM Tris-HCl, pH 8.0, 20 mM NaCl overnight to perform anion exchange chromatography. The soluble protein fraction was applied to a DEAE sepharose column at 4°C for 1 hour and the bound protein eluted from the sepharose by buffer containing 0.5 M NaCl. The eluted proteins were concentrated using a Centricon-50 concentration tube. Finally, the UL97 was subjected to immuno-affinity purification.

### Phosphorylation of Ganciclovir (GCV) by UL97

Sf21 insect cells were infected with wild type or mutant UL97 recombinant baculoviruses at an m.o.i. of 10. After 48 h incubation at 28°C, the culture medium was replaced with fresh medium containing 1.0 mM GCV and [^3^H] GCV (0.55 Mbq, Moravek Radiochemicals, California, USA) After a further 24 hours of incubation, nucleotides were harvested by acid hydrolysis (0.5 M perchloric acid), neutralisation to pH 7.5 (2.5 M KOH, 1.5 M KH_2_PO_4_) and centrifugation (13000 × g). 50 μl aliquots of supernatant were spotted onto 20 mm diameter DEAE-coated DE81 chromatographic paper discs and the monophosphorylated GCV allowed to bind. The discs were washed twice in 3 ml 10 mM ammonium acetate for 10 minutes and twice in 95% ethanol for 10 minutes. After drying 5 ml of sigmafluor was added (Sigma Chemicals, Dorset, UK) and the radioactivity determined by liquid scintillation counting.

### Autophosphorylation of UL97

Analysis of the autophosphorylation of wild type and mutant UL97 protein species was performed by a standard protein kinase assay as previously described (46). Briefly, purified protein was added to 2× protein kinase buffer (100 mM Tris HCl, pH9.0, 20 mM MgCl_2_, 10 μM ATP, 4 mM DTT, 2 M NaCl) and 370 KBq [γ^32^P] ATP (Amersham), made up to 20 μl with SDW and incubated at 37°C. The reactions were terminated by the addition of 2× SDS sample buffer followed by boiling for 3 minutes. The amount of radiolabelled phosphate incorporated by autophosphorylation was analysed by SDS-PAGE and measured using the Biorad Mulitanalyst. The protein kinase assays were initially performed for 2 to 60 minutes to determine the maximum rate of phosphate incorporation, all assays were then performed at the maximum rate which equated to 5 minutes.

### Enzyme kinetic analysis

Enzyme kinetic analysis was performed on the purified wild type and mutant UL97 protein species as above using increasing concentrations of ATP (2 μM to 20 μM). The amount of incorporated radiolabelled phosphate was plotted against the concentration of ATP in a Lineweaver Burke plot to determine the Km for ATP for each UL97 species. The effect of maribavir upon the rate of radiolabelled phosphate incorporation by wild type or mutant UL97 was determined by protein kinase assays at a fixed concentration of maribavir (0.5 μM) as above, or with increasing concentrations of maribavir (0.01 μM to 5.0 μM) to determine the IC_50 _of maribavir for each UL97 species. In order to determine the nature of the inhibition mediated by maribavir, plots of 1/v vs 1/ATP with increasing concentrations of maribavir were constructed. Competitive inhibition was evident if the family of lines cconverged on the y-axis at 1/Vmax. The change in slope caused by the addition of maribavir was used to calculate the Ki using standard enzyme kinetic equations viz.

$$Km^{+I}=Km^{-I}\left(1+\frac{\left[I\right]}{\left[Ki\right]}\right)$$

where *Km ^+I ^*is the apparent *Km *in the presence of the inhibitor, *Km ^-I ^*is the *Km *in the absence of the inhibitor, *Ki *is the dissociation constant of the inhibitor and *[I*] the concentration of the inhibitor.

## Results

### Expression and purification of UL97

**Figure 1:**
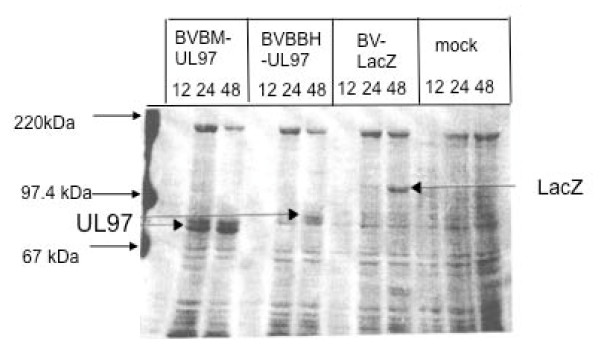
**Coomassie Blue stained SDS-PAGE gel showing the time course of expression of the wild type UL97 protein using either the honey-bee mellitin (BVBM-UL97; lanes 1-3) or the BlueBacHis (BVBBH-UL97; lanes 4-6) recombinant baculovirus system illustrating the higher expression levels observed in the honey-bee mellitin system**. For comparison, a recombinant baculovirus expressing the LacZ gene (BV-LacZ; lanes 7-10) and mock infected insect cells (mock; lanes 10-12) are also shown. Arrows indicate the UL97 and LacZ proteins and molecular weight markers.

**Figure 2:**
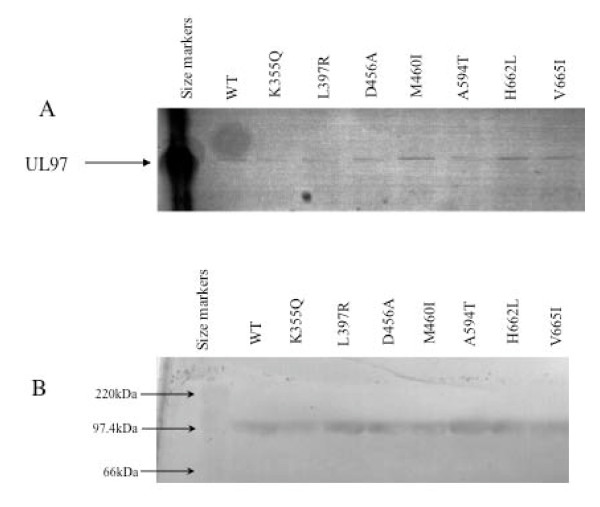
**A) SDS-PAGE analysis of purified wild type and a selection of mutant UL97 proteins used in the study**. B) Western blot analysis showing the comparable expression levels of wild type and mutant UL97 proteins.

Expression of large amounts of wild type and mutant forms of UL97 for the subsequent biochemical analyses was achieved by using two different recombinant baculovirus expression system using either a C-terminal His tag or fusion of the N-terminus with the honey-bee mellitin signal sequence. All mutant constructs were DNA sequenced to ensure that only the desired mutation had been introduced. Expression levels of UL97 were substantially higher in the honey-bee melittin system rather than the his-tag system and so the former system was used for the subsequent studies (Figure 1). Wild type or mutant UL97 proteins were purified using a combination of ammonium sulphate precipitation followed by anion exchange and affinity chromatography. The various UL97 proteins were purified to > 90% homogeneity, were expressed at similar levels and were used at the same protein concentrations for the enzymatic analysis. (Figure 2A and 2B).

### GCV phosphorylation by mutant UL97 proteins

**Figure 3:**
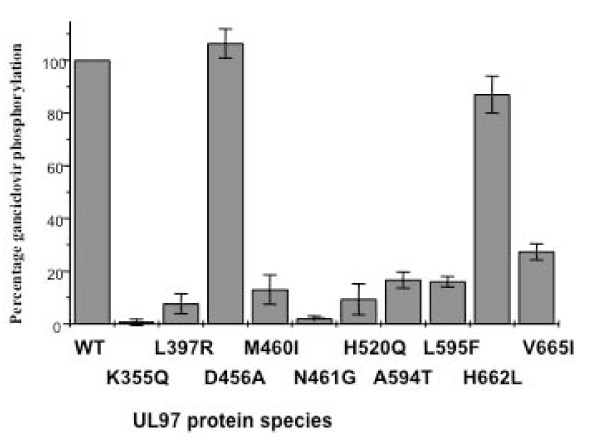
**Ganciclovir (GCV) phosphorylation catalysed by the wild type and UL97 mutant proteins (designated by the amino acid change present)**. Insect cells were infected with the wild type or mutant UL97 expressing baculoviruses at an MOI 10. The medium was supplemented with tritiated GCV at 48 hours. The nucleotides were harvested at 72 hours and bound to DE81 filter paper. The phosphorylated GCV is plotted as percentage phosphorylation compared to the wild type UL97 (set at 100%). Data are the mean +/- one standard deviation of three experiments.

Insect cells were infected with the wild type or mutant UL97-expressing baculoviruses at high multiplicity and after 48 hours, the medium was supplemented with tritiated GCV. The phosphorylation of GCV catalysed by each of the mutant UL97 proteins was compared to wild type GCV phosphorylation (normalised to 100%). The data summarised in Figure 3 show that GCV phosphorylation by the genotypic GCV-resistant UL97 mutants (M460I, H520Q, A594T and L595F) was reduced to between 10% and 20% of the levels of GCV phosphorylation catalysed by the wild type UL97 protein. As expected, mutation at the invariant lysine (K355Q) produced a protein that was unable to phosphorylate GCV. Although mutation of M460 showed a substantial loss of GCV phosphorylation, mutation of the nearby 456 codon had no effect on GCV phosphorylation. Mutations around the His-X-aromatic-hydrophobic motif at codons H662L and V665I showed a reduction in GCV phosphorylation by 12% and 72% of wild type levels respectively. Interestingly, the maribavir-resistant UL97 mutant (L397R) exhibited a significant impairment in GCV phosphorylation (approximately 10% of wild type).

### Kinetic analysis of UL97 autophosphorylation

**Figure 4:**
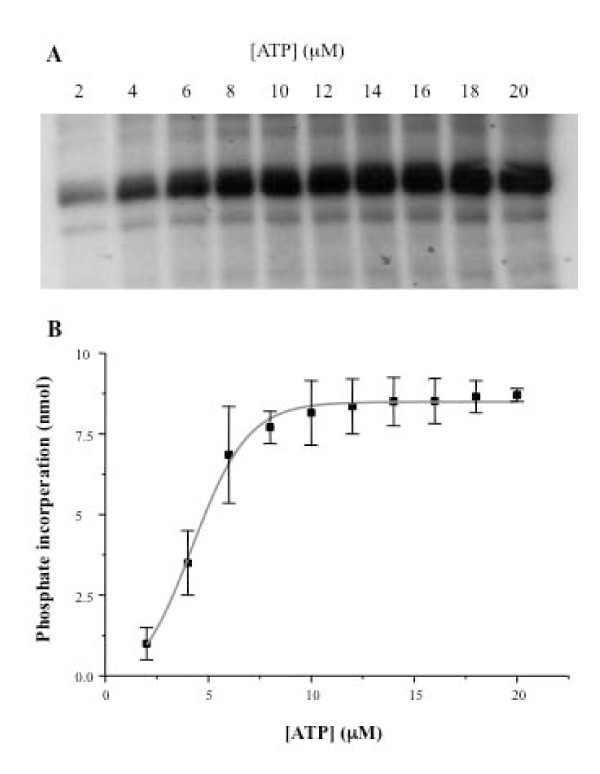
**The kinetics of autophosphorylation of UL97 (panel A)**. The density of the bands was analysed by the BioRad Multianalyst software and then plotted against the corresponding ATP concentration (B). Data shown are for the wild type UL97. Data are the mean +/- one standard deviation of three experiments.

Enzyme kinetic analyses were performed on the wild type and mutant UL97 proteins to calculate the Km for ATP. The purified UL97 proteins were incubated in a protein kinase assay for 5 minutes in constant reaction conditions, but with increasing ATP concentrations (2 μM to 20 μM). The amount of phosphate incorporated was determined (shown for wild type UL97 in Figure 4) and Lineweaver Burke plots used to calculate the Km for ATP. In the case of the wild type UL97, the Km for ATP was 27 ± 2.2 μM and for the GCV resistant mutants, the Km values were as follows: M460I, 28 ± 2 μM; H520Q. 25 ± 2.4 μM; A594T, 40 ± 4.2 μM; L595F, 31 ± 4.5 μM. An alternative analysis which gives the relative efficiency of usage of ATP was performed where Vmax/Km was calculated. These data showed that the GCV resistant mutant proteins were of similar efficiency as the wild type at utilising ATP than the wild type protein except for the M460I mutant which was substantially less efficient (Vmax/Km for wild type = 7.2 s^-1^; M460I = 4.5 s^-1^; H520Q = 8.4 s^-1^; A594T = 5.5 s^-1^; L595F = 6.4 s^-1^).

### Effects of maribavir on the autophosphorylation of wild type and mutant UL97 species

**Figure 5:**
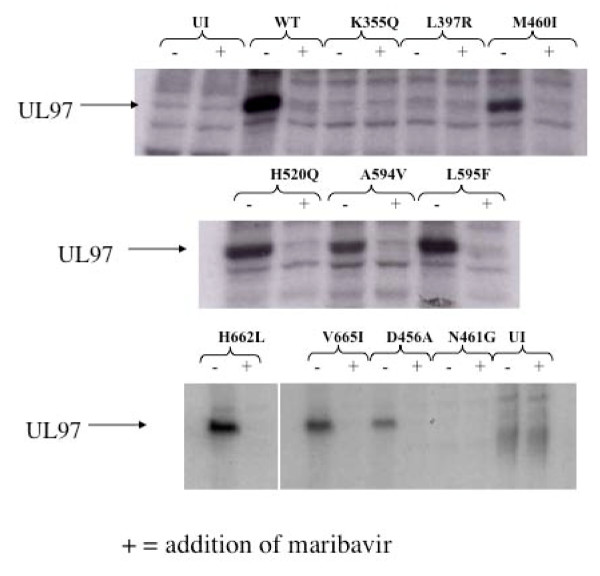
**Effects of maribavir on the autophosphorylation of wild type UL97 and the various mutant proteins**. Autoradiographs showing the Inhibition of autophosphorylation of wild type and mutant UL97 proteins by maribavir. The presence or absence of maribavir (0.5 μM) is indicated by + or-above each lane. UI = uninfected control insect cells.

In the absence of maribavir, the GCV resistant UL97 mutants H520Q, A594T and L595F exhibited autophosphorylation levels equivalent to the wild type UL97 (Figure 5). However, the M460I mutant consistently exhibited approximately 85% of the autophosphorylation levels of the wild type UL97 (p = 0.01). Mutations of the invariant lysine (K355Q) completely negated autophosphorylation, as did mutation of the arginine at amino acid 461. Interestingly, the mutation that confers maribavir resistance to UL97 (L397R) resulted in a reduction in autophosphorylation to approximately 8% of the wild type levels. The remaining mutations within the UL97 gene (D456A, H662L and V665I) did not result in any change in autophosphorylation compared to that catalysed by the wild type UL97.

In the presence of a fixed concentration of maribavir (0.5 μM) the autophosphorylation of every form of UL97 investigated (mutant and wild type) was reduced to below 10% of the total phosphorylation of the wild type UL97 protein (Table 1). As expected, the autophosphorylation of the L397R mutant, which is resistant to maribavir, was unaffected although the absolute autophosphorylation level of this mutant is already substantially reduced (see above).

**Table 1:** Effects of maribavir on the relative levels of autophosphorylation of the wild type and mutant UL97 proteins.

**Ul97 species**	**% autophosphorylation**	
	
	**No Maribavir**	**Plus Maribavir**
Wild type	100	4.5 ± 2.0
K355Q	1.0 ± 0.9	0.5 ± 0.8
L397R	7.8 ± 3.3	7.8 ± 4.5
D456A	104.0 ± 4.0	5.0 ± 1.0
M460V	86 ± 2.0	3.9 ± 2.0
N461G	8.0 ± 1.8	4.2 ± 2.0
H520Q	103.5 ± 3.5	3.8 ± 1.5
A594T	107.0 ± 5.0	2.5 ± 2.5
L595F	100.0 ± 8.0	5.0 ± 1.0
H662L	102.5 ± 4.5	3.0 ± 1.5
V665I	100.5 ± 2.0	3.0 ± 1.5

The data are normalised relative to wild type and show the mean and standard deviation of triplicate analysis.

**Figure 6:**
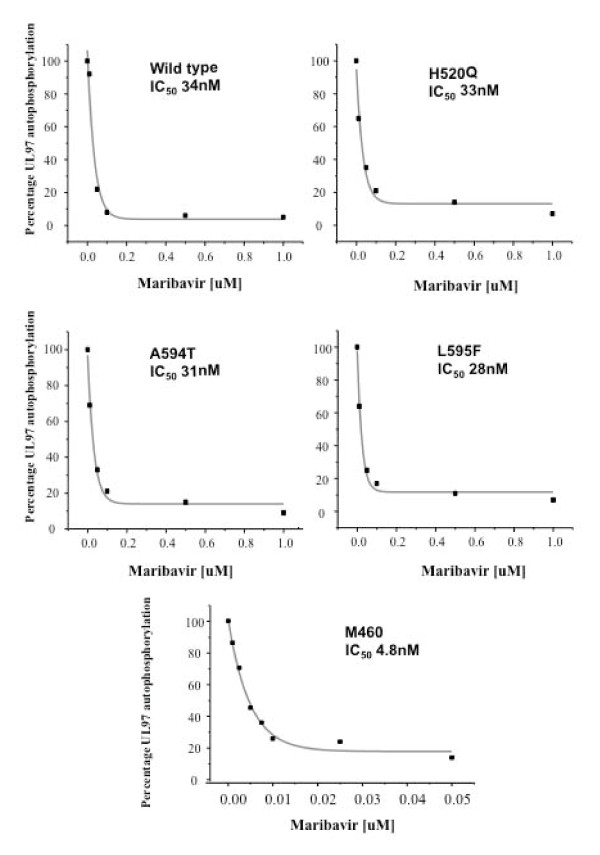
**IC_50 _of maribavir for the wild type and mutant UL97 Proteins**. The wild type and mutant UL97 proteins were subjected to protein kinase assays with varying concentrations of maribavir (0.01-1.0 μM). The autoradiographs were analysed using the BioRad Multianalyst software and UL97 phosphorylation plotted as a percentage of the total phosphorylation in the absence of maribavir. An exponential decay curve was fitted and the IC_50 _of maribavir was determined for each of the proteins. The alteration of x-axis scale of the M460I graph should be noted. The IC_50 _for each species is shown.

Quantitative image analysis of the autoradiographs generated from the protein kinase assays using increasing concentrations of maribavir allowed calculation of the IC50 for each UL97 protein under investigation (Figure 6). The IC_50 _for wild type UL97 was 34 nM, and for the mutants H520Q, A594T, L595F, the D456A, H662L and V665I the IC50 was 33 nM, 31 nM, 28 nM 34 nM, 40 nM and 30 nM respectively. There was no autophosphorylation of the K355Q and N461G mutants hence these proteins were not examined in the presence of maribavir. The maribavir-resistant mutant (L397R) did not exhibit a reduction in autophosphorylation even when drug concentrations were increased to 100 μM. In contrast to the comparable IC_50 _values observed between wild type UL97 and the GCV resistant mutants at amino acids 520, 594 and 595, the M460I mutant was hypersensitive to maribavir with an IC50 of 4.8 nM (Figure 6).

### Mechanism of action of maribavir

**Figure 7:**
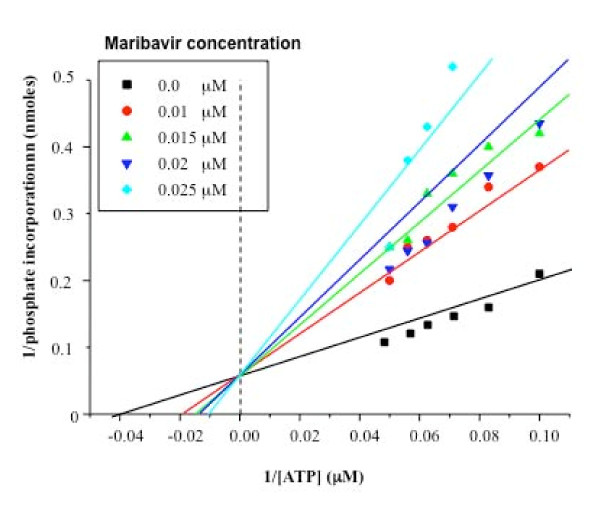
**Competitive inhibition of ATP binding by maribavir**. Protein kinase assays were performed containing increasing concentrations of ATP (2 μM-20 μM) in the absence of maribavir (0.0 μM on the graph) and repeated for increasing maribavir concentrations (0.01 μM, 0.015 μM, 0.2 μM or 0.25 μM). The inverse of the velocity of autophosphorylation was plotted against the corresponding inverse of the [ATP] and the Ki value for maribavir determined using standard enzyme kinetic equations for competitive inhibition.

In order to determine whether maribavir was acting as a competitive inhibitor of ATP, a protein kinase assay was performed using the wild type UL97 protein with increasing concentrations of ATP (2 μM to 20 μM) in the presence of different concentrations of maribavir. The level of UL97 autophosphorylation decreased upon addition of maribavir at each ATP concentration and for each increase in maribavir concentration (Figure 7). The family of lines observed in the Lineweaver Burke plot shown in Figure 7 crossed the y-axis at 1/Vmax and was consistent with maribavir acting as a competitive inhibitor of ATP. The computed Ki for maribavir was 10 ± 0.8 nM.

## Discussion

UL97 is an enigmatic protein which likely possesses a number of roles in the viral life cycle relating to both viral and cellular processes [4-10]. The protein autophosphorylates a number of serine and threonine residues although there is no evidence that it is a substrate for cellular serine/threonine kinses. The availability of a new class of antiviral compounds based upon a benzimidazole core structure that target the kinase activity of UL97 coupled with purified UL97 allowed us to undertake a detailed quantitative biochemical analysis of the functional implications of a spectrum of mutations, including the key mutations giving rise to clinically significant drug resistance against either GCV or maribavir, on both the GCV and protein kinase functions of UL97.

Initially we investigated the function of the mutant UL97 proteins as GCV kinases. Consistent with other studies [46] the results showed that the GCV kinase efficiency of the well characterised GCV resistant mutants of UL97 (M460I, H520Q and A594V and L595F) was between 10-15% of the wild type levels. Mutations in other key domains of UL97 showed a variety of GCV kinase activities. The D456A mutant showed similar GCV phosphorylation levels as the wild type protein despite being part of the highly conserved kinase domain whereas the H662L mutant phosphorylated GCV to 82% of wild type levels. In contrast, the N461G mutant and mutation of the invariant protein kinase lysine at amino acid 355 led to the complete loss of GCV kinase activity while the V665I mutant reduced GCV phosphorylation to 25% of wild type levels. Interestingly, the maribavir resistant mutant L397R was severely debilitated as a GCV kinase (~8% of wild type levels) implying that this mutant would also be cross-resistant to GCV. These data are consistent with recent in vitro studies of this mutant [47].

We next investigated the autophosphorylation of the UL97 proteins. The Km for ATP was similar for both wild type and mutant UL97 proteins (27 μM) and was comparable to that observed in previous studies on U69 protein of HHV-6 [48]. However, when the Vmax/Km were compared most of the GCV resistant UL97 proteins appeared to be similar to the wild type in their utilisation of ATP with the exception of the M460I mutation which was less efficient. This observation is consistent with the observation that the autophosphorylation levels observed for the M460I were reduced compared to wild type and other GCV resistant mutants analysed in this study.

Previous data using UL97 mutants of the invariant lysine at codon 355 and the conserved asparagine at codon 461 has shown that UL97 autophosphorylation is a prerequisite for the phosphorylation of GCV [15]. We have extended these analyses to include a substantial range of additional mutants and investigated the effects of maribavir on each mutant species. Interestingly, with the exception of mutations at essential amino acids (L355 and N461) the majority of GCV resistant mutants showed autophosphorylation levels comparable to the wild type. The only exceptions were the M460I mutation which exhibited autophosphorylation at 83% of wild type levels and the maribavir resistant mutant (L397R) where autophosphorylation was reduced to ~10% of wild type levels. Thus, certain UL97 mutations can possess both high level protein and GCV kinase activity ((H662L, D456A) or high level protein kinase function and low level GCV kinase activity (M460I, A594T, L595F, H520Q). However, only the L397R mutation exhibited both low protein and low GCV kinase activity. Addition of maribavir resulted in a substantial reduction in the autophosphorylation of wild type and all mutant UL97 proteins except for mutations that either negated protein kinase function (K355Q) or contained the maribavir resistance mutation (L397R). Based upon data for GCV resistant UL97 mutants in vivo [35], the low level of autophosphorylation associated with the L397 mutant would result in this mutant being substantially less fit than wild type virus in vivo. It will be interesting to perform similar autophosphorylation analysis on the recently described additional maribavir resistant mutants [42,43] and extend these studies to determine relative fitness differences between these maribavir resistant viruses and their wild type counterparts.

The IC_50 _for maribavir of all the UL97 species were very similar with a mean value of 33 nM (+/- 12 nM). Importantly, each of the phenotypic GCV-resistant mutants exhibited the equivalent sensitivity to maribavir as the wild type UL97. However, the IC_50 _of maribavir for the M460I mutant (4.8 nM) showed that this mutant was approximately four times more sensitive to the drug. This observation may reflect the position of the 460 codon in the conserved protein kinase consensus sequence HRDLKXXN (amino acids 454-461 in UL97) that comprises the catalytic loop. Unfortunately, the N461G mutation exhibited a total loss in autophosphorylation and so further investigations were precluded. The fact that the L397R mutation (and the other more recently described mutations [42]) reside in the protein kinase subdomain III, which is involved in the stabilisation of the interaction between the invariant lysine and the α- and β- phosphates of the ATP, suggests that maribavir may interfere with this interaction. We showed that the inhibition by maribavir was alleviated at high concentrations of ATP and increasing concentrations of maribavir reduced the incorporation of radiolabelled phosphate in the UL97 autophosphorylation assay. When the Lineweaver-Burke plots were constructed, the resulting family of lines converged on the Y-axis at approximately 1/V_max_. These observations were consistent with maribavir acting as a competitive inhibitor of ATP with a computed Ki of 10 nM.

In conclusion, we have shown that GCV resistant mutations in the UL97 kinase do not affect the Km of the enzyme for ATP but that the UL97 M460 mutation confers hypersensitivity to maribavir. Maribavir is a potent competetitive inhibitor of ATP and a mutation at L397 that yields maribavir resistance substantially reduces both the GCV kinase and protein kinase activity of UL97 meaning that maribavir resistant strains of HCMV will have to be treated with foscarnet or cidofovir.

## Competing interests

VCE has acted on advisory boards and as a speaker for Roche Pharmaceuticals. VCE has also acted on advisory boards for Viropharma. No other potential conflicts of interest exist.

## Authors' contributions

VCE designed the experimental approach, wrote the manuscript and supervised the work. CASL performed the experimental work, helped design the experiments and contributed to the writing of the manuscript. All authors read and approved the final manuscript.
